# Particle Physics and Cosmology Intertwined

**DOI:** 10.3390/e26020110

**Published:** 2024-01-25

**Authors:** Pran Nath

**Affiliations:** Department of Physics, Northeastern University, Boston, MA 02115-5000, USA; p.nath@northeastern.edu

**Keywords:** particle physics, cosmology

## Abstract

While the standard model accurately describes data at the electroweak scale without the inclusion of gravity, beyond the standard model, physics is increasingly intertwined with gravitational phenomena and cosmology. Thus, the gravity-mediated breaking of supersymmetry in supergravity models leads to sparticle masses, which are gravitational in origin, observable at TeV scales and testable at the LHC, and supergravity also provides a candidate for dark matter, a possible framework for inflationary models and for models of dark energy. Further, extended supergravity models and string and D-brane models contain hidden sectors, some of which may be feebly coupled to the visible sector, resulting in heat exchange between the visible and hidden sectors. Because of the couplings between the sectors, both particle physics and cosmology are affected. The above implies that particle physics and cosmology are intrinsically intertwined in the resolution of essentially all of the cosmological phenomena, such as dark matter and dark energy, and in the resolution of cosmological puzzles, such as the Hubble tension and the EDGES anomaly. Here, we give a brief overview of the intertwining and its implications for the discovery of sparticles, as well as the resolution of cosmological anomalies and the identification of dark matter and dark energy as major challenges for the coming decades.

## 1. Introduction

This article is a contribution to Paul Frampton’s 80th birthday volume, marking his over five decades of contributions as a prolific researcher to theoretical physics. He is one of the few theoretical physicists who recognized early on that there is no boundary between particle physics and cosmology and contributed freely to each in good measure. His prominent works include those in particle theory, such as those related to physics beyond the standard model and anomaly cancellations in higher dimensions, and in cosmology, such as those focusing on non-standard cosmological models and black-hole physics. Since particle physics and cosmology are the two major areas of his work, this paper elaborates on the progressive intertwining of the fields of particle physics and cosmology over the past several decades from the author’s own perspective.

For a long period of time, up to and including the period of the emergence of the standard model [1,2,3,4,5,6,7] and its tests, it was largely accepted that gravity could be ignored in phenomena related to particle physics. The contrary, of course, was not true, as particle physics was already known to be central to a variety of astrophysical phenomena, such as the Chandrasekhar limit [8] and the synthesis of elements in the work of B^2^FH [9] and Peebles [10]. For particle physics, gravity became more relevant with the emergence of supersymmetry, supergravity, and strings. Further, supergravity models with the gravity-mediated breaking of supersymmetry lead to soft terms that allow for the radiative breaking of electroweak symmetry and predict sparticles observable at colliders. There is another aspect of supergravity and strings that has a direct impact on particle physics. In extended supergravity, string, and D-brane models, one finds hidden sectors that can couple feebly with the visible sector and affect particle physics phenomena observable at colliders and that also have implications for cosmology, as they can provide candidates for inflation, dark matter, and dark energy. Thus, with the emergence of supergravity and strings, a deeper connection between particle physics and cosmology has emerged. Of course, one hopes that particle physics and cosmology are parts of strings, and significant literature exists on the particle-physics–string connection (see, e.g., [11,12,13,14,15] and references therein) and on the cosmology–string connection (see, e.g., [16,17,18] and references therein).

In this paper, we will focus on the intertwining of particle physics and cosmology. As noted above, this intertwining has occurred on two fronts: first, in supergravity models with gravity-mediated breaking, the sparticle spectra are direct evidence that gravitational interactions are at work even at the scale of electroweak physics. Further, supergravity models with R-parity conservation lead to a candidate for dark matter, specifically a neutralino [19]. Most often, it turns out to be the lightest supersymmetric particle in the radiative breaking of electroweak symmetry [20]. The neutralino as dark matter importantly enters simulations of cosmological evolution. At the same time, supergravity provides models for the inflationary expansion of the universe. Second, as noted above, in extended supergravity and string models, one finds hidden sectors, some of which may be feebly coupled to the visible sector. Typically, the hidden sectors and the visible sector will have different temperatures, but they have heat exchange, which requires the synchronous evolution of the two sectors, thus intertwining the two and affecting both particle physics and cosmology. The outline of the rest of the paper is as follows. In Section 2, we will discuss the implications of the gravity-mediated breaking of supergravity at low energy, and Section 3 focuses on the intertwining of particle physics and cosmology via hidden sectors.

## 2. Gravitational Imprint on Particle Physics at the Electroweak Scale

As noted above, until the advent of Sugra, it was the prevalent view that gravity did not have much of a role in particle physics models. However, with the advent of supergravity grand unification [21,22], where supersymmetry is broken in the hidden sector and communicated to the visible sector through gravitational interactions, one finds that soft breaking terms are dependent on gravitational interactions [21,23,24]. Thus, the soft mass of scalars in the visible sector $m_{s}\propto \kappa m^{2}$, where $\kappa =\sqrt{8\pi G_{N}}$ and $G_{N}$ is Newton’s constant, and *m* is an intermediate hidden sector mass. Here, with $m∼10^{10}$ and $M_{Pl}=\kappa^{-1}=2.43\times 10^{18}$ GeV (in natural units: ℏ=c=1), one finds $m_{s}$ to be of electroweak size. Since sparticle masses are controlled by the soft susy scale, the discovery of sparticles would be a signature indicating that gravity has a role in low -energy physics. This would very much be akin to the discovery that the *W* and *Z* bosons are a reflection of $SU{\left(2\right)}_{L}\times U{\left(1\right)}_{Y}$ unification. It is notable that the soft terms are also responsible for generating the spontaneous breaking of electroweak symmetry [21,25]. An indication that some of the sparticles may be low-lying comes from the g−2 data from Fermilab [26], which point to a deviation from the standard-model prediction of about 4σ. An attractive proposition is that the deviation arises from light-sparticle exchange, specifically light charginos and light sleptons (see, e.g., [27,28,29] and the references therein), a deviation that was predicted quite a while ago [30]. However, a word of caution is in order, in that the lattice analysis [31] for the hadronic vacuum polarization contribution gives a smaller deviation from the standard model than the conventional result, where the hadronic vacuum polarization contribution is computed using $e^{+}e^{-}\to \pi^{+}\pi^{-}$ data. Thus, further work is needed to reconcile the lattice analysis with the conventional result on the hadronic polarization contribution before drawing any definitive conclusions.

## 3. Hidden Sectors Intertwine Particle Physics and Cosmology

As already noted, in a variety of models beyond standard-model physics, which include extended supergravity models, string models, and extra-dimension models, one has hidden sectors. While these sectors are neutral under the standard-model gauge group, they may interact with the visible sectors via feeble interactions. Such feeble interactions can occur via a variety of portals, which include the Higgs portal [32], kinetic energy portal [33,34], Stueckelberg mass-mixing portal [35,36], kinetic-mass-mixing portal [37], and Stueckelberg–Higgs portal [38], as well as possible higher-dimensional operators. The hidden sectors could be endowed with gauge fields, as well as with matter. At the reheat temperature, the hidden sectors and the visible sector would, in general, lie in different heat baths. However, because of the feeble interactions between the sectors, there will be heat exchange between the visible and hidden sectors, and thus, their thermal evolution will be correlated. The evolution of the relative temperatures of the two sectors then depends on the initial conditions, specifically on the ratio $\xi \left(T\right)=T_{h}/T$ at the reheat temperature, where $T_{h}$ is the hidden-sector temperature, and *T* is the visible-sector temperature. The Boltzmann equations governing the evolution of the visible and hidden sectors are coupled and involve the evolution equation for ξ(T). Such an equation was derived in [39,40,41] and applied in a variety of settings in [42], consistent with all experimental constraints on hidden-sector matter from terrestrial and astrophysical data [43]. It is found that hidden sectors can affect observable phenomena in the visible sector, such as the density of thermal relics. Hidden sectors provide candidates for dark matter and dark energy and help resolve cosmological anomalies intertwining particle physics and cosmological phenomena. We discuss some of these topics in further detail below.

Green-Schwarz [44] found that in the low-energy limit of Type I strings, the kinetic energy of the two-tensor $B_{MN}$ of a 10D supergravity multiplet has Yang–Mills and Lorentz-group Chern–Simons terms (indicated by superscripts Y and L) so that $\partial_{[P}B_{MN]}\to \partial_{[P}B_{MN]}+\omega_{PMN}^{\left(Y\right)}-\omega_{PMN}^{\left(L\right)}$, where M,N, and *P* are 10-dimensional indices. The inclusion of the Chern–Simons terms fully requires that one extend the 10D Sugra Lagrangian to order $O{\left(\kappa \right)}^{2}$. This was accomplished subsequent to Green-Schwarz’s work in [45] (for related works, see [46,47,48]). Dimensional reduction to 4D with a vacuum expectation value for the internal-gauge-field strength, $\langle F_{ij}\rangle \neq 0$ (where the indices are for the six-dimensional compact manifold), leads to $\partial_{\mu }B_{ij}+A_{\mu }F_{ij}+\;\cdots \;∼\;\partial_{\mu }\sigma +mA_{\mu }$ (μ in an index for four-dimensional Minkowskian space–time), where the internal components $B_{ij}$ give the pseudo-scalar σ, and *m* arises from $<F_{ij}>$, which is a topological quantity, related to the Chern numbers of the gauge bundle. Thus, $A_{\mu }$ and σ have a Stueckelberg coupling of the form $A_{\mu }\partial^{\mu }\sigma$. This provides the inspiration for building BSM models with the Stueckelberg mechanism [35,36,49,50,51]. Specifically, this allows for the possibility of writing effective theories with gauge-invariant mass terms. For the case of a single U(1) gauge field $A_{\mu }$, one may write a gauge-invariant mass term by letting $A_{\mu }\to A_{\mu }+\frac{1}{m}\partial_{\mu }\sigma$, where the gauge transformations are defined so that $\delta A_{\mu }=\partial_{\mu }\lambda$ and δσ=−mλ. In this case, σ’s role is akin to that of the longitudinal component of a massive vector. The above technique also allows one to generate invariant mass mixing between two U(1) gauge fields. Thus, consider two gauge groups $U{\left(1\right)}_{X}$ and $U{\left(1\right)}_{Y}$ with gauge fields $A_{\mu }$ and $B_{\mu }$ and an axionic field σ. In this case, we can write a mass term ${\left(m_{1}A_{\mu }+m_{2}B_{\mu }+\partial_{\mu }\sigma \right)}^{2}$ that is invariant under $\delta_{x}A_{\mu }=\partial_{\mu }\lambda_{x}$, $\delta_{x}\sigma =-m_{1}\lambda_{x}$ for $U{\left(1\right)}_{X}$, and $\delta_{y}B_{\mu }=\partial_{\mu }\lambda_{y}$, $\delta_{y}\sigma =-m_{2}\lambda_{y}$ for $U{\left(1\right)}_{y}$. One of the interesting phenomena associated with effective gauge theories with gauge-invariant mass terms is that they generate millicharges when coupled to matter fields [35,37,49,52]. We will return to this feature of the Stueckelberg mass-mixing terms when we discuss the EDGES anomaly.

Hubble tension: Currently, there exists a discrepancy between the measured value of the Hubble parameter $H_{0}$ for low redshifts (z<1) and high redshifts (z>1000). Thus, for (z<1), an analysis of data from Cepheids and SNIa gives [53] $H_{0}=\left(73.04\pm 1.04\right)\;\mathrm{km}/s/\mathrm{Mpc}.$ On the other hand, an analysis based on the ΛCDM model by the SH0ES Collaboration [53] using data from the cosmic microwave background (CMB), Baryon Acoustic Oscillations (BAOs), and Big Bang Nucleosynthesis (BBN) determines the Hubble parameter at high *z* to be [54] $H_{0}=\left(67.4\pm 0.5\right)\;\mathrm{km}/s/\mathrm{Mpc}.$ This indicates a 5σ-level tension between the low-*z* and the high-*z* measurements. There is a significant amount of literature attempting to resolve this puzzle, at least partially, and recent reviews include [55,56]. One simple approach is introducing extra relativistic degrees of freedom during the period of recombination, which increases the magnitude of $H_{0}$, which helps alleviate the tension. Models using this idea introduce extra particles, such as the $Z^{\prime }$ of an extra U(1) gauge field that decays to neutrinos [57,58], or utilize other particles, such as the majoron [59,60]. The inclusion of extra degrees of freedom, however, must be consistent with the BBN constraints, which are sensitive to the addition of massless degrees of freedom. Thus, the standard-model prediction of $N_{\mathrm{eff}}^{\mathrm{SM}}≃3.046$ [61] is consistent with the synthesis of light elements, and the introduction of new degrees of freedom must maintain this successful standard-model prediction. The above indicates that the extra degrees of freedom should emerge only beyond the BBN time and in the time frame of the recombination epoch. It is noted that new degrees of freedom are also constrained by the CMB data, as given by the Planck analysis [54].

A cosmologically consistent model based on the Stueckelberg extension of the SM with a hidden sector was proposed in [62] for alleviating the Hubble tension. The model is cosmologically consistent since the analysis is based on a consistent thermal evolution of the visible and hidden sectors, taking account of the thermal exchange between the two sectors. In addition to dark fermions and dark photons, the model also contains a massless pseudo-scalar particle field ϕ and a massive long-lived scalar field *s*. The fields ϕ and *s* have interactions only in the dark sector, with no interactions with the standard-model fields. The decay of the scalar field occurs after BBN, close to the recombination time, via the decay s→ϕϕ, which provides the extra degrees of freedom needed to alleviate the Hubble tension. It should be noted that the full resolution of the Hubble tension would require going beyond providing new degrees of freedom and would involve a fit to all of the CMB data that are consistent with all cosmological and particle physics constraints. For some recent related work on the Hubble tension, see [63,64,65,66,67,68].

EDGES anomaly: The 21 cm line plays an important role in the analysis of physics during the dark ages and the cosmic dawn in the evolution of the early universe. The 21 cm line arises from the spin transition from the triplet state to the singlet state and vice versa in the ground state of neutral hydrogen. The relative abundance of the triplet and singlet states defines the spin temperature $T_{s}$ (and $T_{B}=T_{s}$) of hydrogen gas and is given by $n_{1}/n_{0}=3e^{-T_{*}/T_{s}}$, where 3 is the ratio of the spin degrees of freedom for the triplet versus the singlet state, $T_{*}$ is defined by $\Delta E=kT_{*}$, where ΔE=1420 MHz is the energy difference at rest between the two spin states, and $T_{*}\equiv \frac{hc}{k\lambda_{21\mathrm{cm}}}=0.068\;K$. EDGES (the Experiment to Detect the Global Epoch of Reionization Signature) reported an absorption profile centered at the frequency ν=78 MHz in the sky-averaged spectrum.The quantity of interest is the brightness temperature $T_{21}$ of the 21 cm line defined by $T_{21}\left(z\right)=\left(T_{s}-T_{\gamma }\right)\left(1-e^{-\tau }\right)/\left(1+z\right)$, where $T_{\gamma }\left(z\right)$ is the photon temperature at redshift *z*, and τ is the optical depth for the transition. The analysis of Bowman et al. [69] finds (see, however, reference [70] on concerns regarding the modeling of data) that at redshift z∼17, $T_{21}=-500_{-500}^{+200}$ mK at 99% C.L. On the other hand, the analysis of [71] based on the ΛCDM model gives a $T_{21}$ of around −230 mK, which shows that the EDGES result is a 3.8σ deviation away from that of the standard cosmological paradigm.

The EDGES anomaly is not yet confirmed, but pending its possible confirmation, it is of interest to investigate what possible explanations there might be. In fact, several mechanisms have already been proposed to explain the 3.8σ anomaly [72,73,74,75,76,77,78,79,80,81,82,83,84,85,86,87,88]. A list of some of the prominent possibilities consists of the following: (1) astrophysical phenomena, such as radiation from stars and star remnants; (2) a hotter CMB background radiation temperature than expected; (3) cooler baryons than what ΛCDM predicts; (4) the modification of cosmological evolution: the inclusion of dark energy such as Chapligin gas. Of the above, there appears to be a leaning toward baryon cooling, and there is a substantial amount of work in this area following the earlier works of [89] and Barkana [78]. Specifically, it was pointed out in [78] that the observed anomaly could be explained if the baryons were cooled down by roughly 3 K. Here, one assumes that a small percentage of DM (∼0.3%) is millicharged and that baryons become cooler through Rutherford scattering from the colder dark matter. As mentioned earlier, precisely such a possibility occurs via Stueckelberg mass mixing if we assume that one of the gauge fields $U{\left(1\right)}_{Y}$ is the hyper-charge gauge field, while $U{\left(1\right)}_{X}$ is a hidden-sector field, and the millicharged dark matter resides in the hidden sector, while the rest of the dark matter could be WIMPS. Within this framework, a cosmologically consistent analysis of a string-inspired millicharged model was proposed in [90], where a detailed fit to the data is possibly consistent within a high-scale model. For some recent work on the EDGES anomaly, see [91,92,93,94].

Inflation: As is well known, the problems associated with the Big Bang, such as the flatness, horizon, and monopole problems, are resolved in inflationary models. In models of this type, quantum fluctuations at the horizon exit encode information regarding the characteristics of the inflationary model that can be extracted from the cosmic microwave background (CMB) radiation anisotropy [95,96,97,98,99]. In fact, data from the Planck experiment [100,101,102] have already put stringent bounds on inflationary models, eliminating some. A model proposed in [103,104] is based on an axionic field with a potential of the form $V\left(a\right)=\Lambda^{4}\left(1+\cos(\frac{a}{f})\right)$, where *a* is the axion field and *f* is the axion decay constant. However, for the simple model above to hold, the Planck data require $f>10M_{Pl}$, which is undesirable since string theory indicates that *f* lies below $M_{Pl}$ [105,106]. However, a reduction in *f* turns out to be a non-trivial issue. The techniques used to resolve this issue include the alignment mechanism [107,108], n-flation, coherent enhancement [109], and models using shift symmetry (for a review and more references, see [110,111]).

We mention another inflation model, which is based on an axion landscape with a U(1) symmetry [112]. This model involves *m* pairs of chiral fields, and the fields in each pair are oppositely charged under the same U(1) symmetry. Our nomenclature is such that we label the pseudo-scalar component of each field as an axion and the corresponding real part as a saxion. Since the model has only U(1) global symmetry, the breaking of the global symmetry leads to just one pseudo-Nambu–Goldstone boson (PNGB), and the remaining pseudo-scalars are not PNGBs. Thus, the superpotential of the model consists of a part that is invariant under the U(1) global symmetry and a U(1)-symmetry-breaking part that simulates instanton effects. The analysis of this work shows that the potential contains a fast-roll–slow-roll-splitting mechanism, which splits the axion potential into fast-roll and slow-roll parts, where the fields entering the fast roll are eliminated early on, leaving the slow-roll part, which involves a single axion field that drives inflation. Here, under the constraints of stabilized saxions, one finds inflation models with $f<M_{Pl}$ to be consistent with the Planck data. Similar results are found in the Dirac–Born–Infeld-based models [113].

Dark energy: One of the most outstanding puzzles of both particle physics and of cosmology is dark energy, which constitutes about 70% of the energy budget of the universe and is responsible for the accelerated expansion of the universe. Dark energy is characterized by negative pressure such that *w*, defined by w=p/ρ, where *p* is the pressure and ρ is the energy density for dark energy, must satisfy w<−1/3. The CMB and the BAO data fit well with a cosmological constant Λ that corresponds to w=−1. Thus, the Planck Collaboration [54] gives w=−1.03±0.03, consistent with the cosmological constant. There are two puzzles connected with dark energy. First, the use of the cosmological constant appears artificial, and it is desirable to replace it with a dynamical field, i.e., a so-called quintessence field (for a review, see [114]), which, at late times, can generate accelerated expansion similar to that given by Λ. The second problem relates to the very small size of the cosmological constant, which is not automatically resolved by simply replacing Λ with a dynamical field. The extreme fine-tuning needed in a particle physics model to get to the size of Λ requires a new idea, such as vacuum selection in a landscape with a large number of possible allowed vacua [115], for instance, those available in string theory. In any case, it is an example of the extreme intertwined nature of cosmology and particle physics. However, finding a quintessence solution that replaces Λ and is consistent with all of the CMB data is itself progress. Regarding experimental measurement of w=−1.03±0.03, if more accurate data in the future give w>−1, it would point to something like quintessence, while w<−1 would indicate phantom energy and an entirely new sector.

## 4. Conclusions

In conclusion, it is clear that particle physics and cosmology are deeply intertwined, and in the future, models of physics beyond the standard model will be increasingly constrained by particle physics experiments as well as by astrophysical data. We congratulate Paul for his notable contributions in the twin fields and wish him many productive years of contributions for the future.

## Data Availability

Data is contained within the article.
